# Telocytes in the Spleen

**DOI:** 10.1371/journal.pone.0138851

**Published:** 2015-09-23

**Authors:** Yuqiao Chang, Cixia Li, Li Gan, He Li, Zhikun Guo

**Affiliations:** 1 Henan Key Laboratory of Medical Tissue Regeneration, Xinxiang Medical University, Xinxiang 453003, P.R. China; 2 Department of Human Anatomy and Embryology, Tongji Medical College, Huazhong University of Science and Technology, Wuhan 430030, P.R. China; Brigham and Women's Hospital, Harvard Medical School, UNITED STATES

## Abstract

Telocytes, a novel type of interstitial cells with very long and thin prolongations, have been identified in many organs in mammals. At present, the ultrastructural, immunocytochemical and electrophysiological properties of telocytes in multiple organs have been understood. However, telocytes in spleen, especially their roles in spleen have not been reported. The aim of this study was to investigate the ultrastructure, distribution and immunophenotypes of splenic telocytes. Rat spleen was harvested for the ultrastructure analysis by transmission electron microscopy (TEM). The primary culture of telocytes was performed after combined enzymatic digestion. The characteristic morphology was analyzed by a scanning electron microscopy (SEM). It was shown that telocytes displayed a piriform/spindle/triangular shape with long and slender telopods and extremely long prolongation contracting with surrounding cells in the spleen. Their dynamic profiles of cytoplasmic separation were recorded by the Live Cell Imaging System. The length of telopods was mostly distributing in 20–30 μm, in accordance with normal distribution. Most telocytes had three or two telopods (28.71% and 22.58% respectively). Immunostaining indicated that these cells were positive for vimentin, CD34, nanog and sca-1, but negative for c-kit. These data prove the existence of telocytes in the spleen, which may serve as the experimental base for exploring their roles in the spleen.

## Introduction

As a new type of interstitial cells, telocytes(TCs) were detected in various tissues/organs in mammals, such as heart [1,2], skeletal muscle [3], respiratory system [4,5], urinary system [6,7], meninges and choroid plexus [8], skin [9], limbus and uvea of eye [10]. Telocytes were characterized by specific ultrastructural features of long and thin cell prolongations and dilated thick regions [11,12]. Through their long prolongations, they kept touch with nerve endings, blood vessels and different types of resident cells [13], forming a three-dimensional network to maintain organism homeostasis.

Hinescu *et al*. reported that TCs represented about 1–1.5% of the atrial myocardial volume and presumed their potential role in interstitium, such as players in pacemaking, intermediates between intrinsic nerve fibers and myocytes, a paracrine and/or juxtacrine source of signal molecules [14]. Gherghiceanu insisted that TCs formed an interstitial network that played a very important role in heart development, renewal and repair [15]. Intramyocardial transplantation of TCs had the potential to reduce myocardial infarction and to improve cardiac function in rats [16]. TCs were also identified in stem cell niches in the heart, skeletal muscle and skin, suggesting their potential role in regeneration and repair of injured tissues [17–19].

Up to now, the presence of TCs in the spleen has not yet been demonstrated, especially their roles in the spleen has been left unknown. The major aim of this study is to provide extensive direct evidence for TCs in the spleen and to detect their immunophenotypes in order to explore the role of TCs in spleen.

## Materials and Methods

### Animals

Four male Sprague-Dawley rats(8-week-old, 220±3.2 g) and twenty neonatal Sprague-Dawley rats (1–3 day-old, 7.2±0.31 g) were utilized in this study. Animal treatment was performed according to the guidelines of *The Ministry of Science and Technology of the People’s Republic of China [(2006)398]* and approved by the Xinxiang Medical University Animal Care Committee (No 030032).

### Transmission electron microscopy

Male adult rats were euthanized with chloral hydrate (10%, 0.1 ml/100 g) by peritoneal injection. The spleens were harvested and cut into small pieces about 1 mm^3^ and immediately immersed in a solution of 4% glutaraldehyde (pH 7.3, 4°C). Fixed samples were washed in phosphate buffer, and were fixed in 1% osmium tetroxide (Polysciences Inc. Warrington, USA) for 1 hr. Samples were then rinsed extensively in 0.1 M cacodylate buffer. Following several rinses, samples were dehydrated in a graded series of ethanol and were embedded in Epon 812 resin (Ted Pella Inc. California, USA). The embedded samples were heat dried at different temperatures (37°C for overnight, 45°C for 12 hrs and 60°C for 48 hrs). Then sections of 50 nm were cut with a Leica Ultracut UCT ultramicrotome (Leica Microsystems Inc, LKB-II, Germany), stained with 3% solution of uranyl acetate and lead citrate, and mounted on formvar-coated 50 mesh grids. Digital pictures were obtained and observed at an acceleration voltage of 80 kV, in JEOL JEM-1230 (Japan) electron microscope.

### Isolation and cell culture of splenic telocytes

Samples from neonatal rat spleens were harvested under sterile conditions and mechanically minced into small pieces of blocks about 1 mm^3^ after washing with sterile phosphate-buffered saline (PBS), then incubated at 37°C for 6 min with 0.1% collagenase I/0.125% trypsin (Sigma-Aldrich, St. Louis, MO, USA) in PBS. The suspension was collected and added into an equal volume of dulbecco minimum essential medium–low glucose (DMEM-LG), supplemented with 10% fetal calf serum (Gibco, New York, USA) to inactivate the role of combined enzymes. This process was repeated for 2–3 times until the tissue blocks were completely digested. And then, the suspension was centrifuged at 1,000 r/min for 5 min. The cellular pellet was re-suspended in DMEM-LG culture medium supplemented with 10% fetal calf serum, 100 U/ml penicillin and 100 mg/ml streptomycin (Sigma-Aldrich). Cell density was counted in a haemocytometer and viability was assessed by Trypan blue staining. The cells were placed in 24-well plates and cultured in a CO_2_ incubator at 37°C. After 45 min, suspensions were plated in 24-well plates to remove fibroblasts. Culture medium was changed every 48 hrs. The morphology of TCs were observed and imaged with a phase-contrast microscope (Diavert, Leica, Wetzlar, Germany).

### Scanning electron microscopy

TCs were fixed in 3% glutaraldehyde in PBS (pH 7.2) for 30 min at room temperature, then dehydrated through graded ethanol (30%, 50%, 70%, 85% and 90%) and preprocessed with vacuum deposition of gold plating, which were imaged by a FEI Quanta200 environmental scanning electron microscope (FEI Co., Hillsboro, OR, USA).

### Dynamic morphology analysis

TCs were plated in dish and cultured in CO2 incubator. When cells reached 50%-60% confluence, the medium was changed with DMEM-LG medium containing 0.05 mg/L 4', 6—diamidino-2-phenylindole (DAPI) in a CO2 incubator at 37°C about 40 min, and then the supernatant was discarded to remove the unbinding of DAPI, subsequently washed with PBS. TCs cultured in complete DMEM-LG medium were detected under a Live Cell Imaging System (xcellence, Olympus, Japan). Parameters were set as follows: 1 image/2 min, duration time 24 hrs.

### Immunoflurescence

The cultured cells were fixed in 4% paraformaldehyde for 20 min at room temperature, subsequently washed with PBS and treated with 0.3% Triton X-100(Sigma) in PBS for 15 min. Then the samples were incubated with PBS containing 2% bovine serum albumin (BSA) at 37°C for 10 min. Incubation with the primary antibodies(mouse anti-c-kit, Santa Cruz, 1:200; Rabbit anti-vimentin, Abcam, 1:100; Rabbit anti-CD34, Bioss, 1:300; Rabbit anti-Nanog, Santa Cruz, 1:100; Rabbit anti-sca-1, Millipore,1:200) was performed at 4°C overnight. The samples were subsequently incubated FITC- or Cy3-conjugated secondary antibodies (Beyotime). The nuclei were counterstained with 1μg /ml DAPI (Roche). Negative controls were obtained by following all the same protocol but the primary antibodies. All experiments were performed in triplicate.

### Statistical analysis

Data were presented as mean ± SD. One-way (ANOVA) and the LSD tests were applied using SPSS version 19.0. *P* < 0.05 was considered statistically significant.

## Results

### Transmission electron microscopy

**Fig 1:**
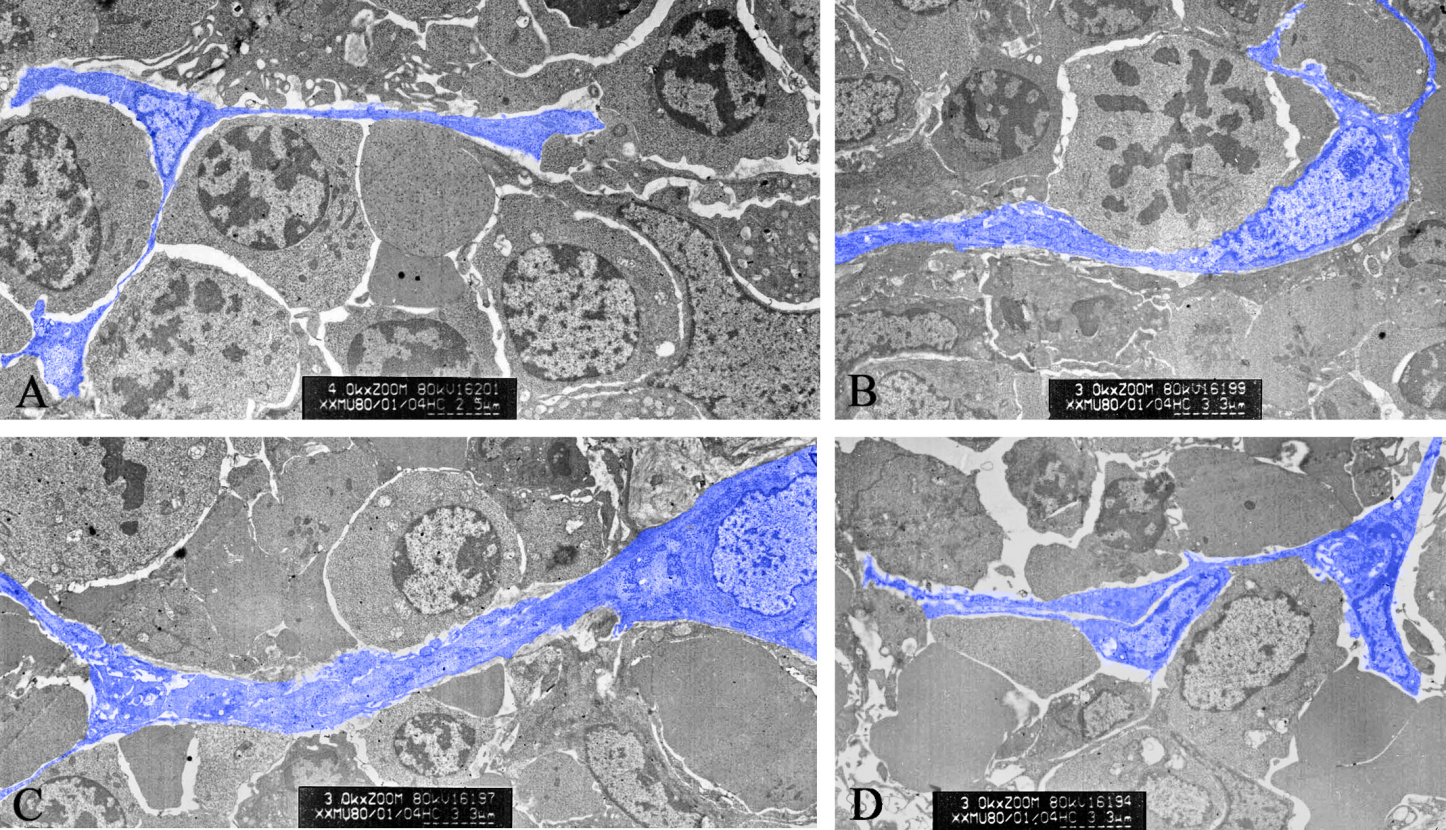
Ultrastructure of splenic TCs under transmission electron microscopy. **A.** A TC with telopodes (Tps) in close contact with lymphocytes. **B.** A TC with Tps embraces with leukocyte. **C.** Tps with two branches distribution. **D.** Clusters of TCs between leukocytes and red blood cells in spleen.

In spleen, TCs with long telopode (TP) were seen clearly in red pulp. They connected with leukocytes and red blood cells through TP. TPs were detected mostly basing on their discontinuous segments with alternation of podom and podomer by TEM. TCs embraced with one or more leukocytes, forming a network structure or labyrinthine structure (Fig 1). The nucleus of TCs appeared in irregular shapes and had condensed heterochromatin combined with the nuclear envelope. Clusters of TCs between leukocytes and red blood cells, connected with each other closely through their TPs (Fig 1).

### Isolated and cultured TCs

**Fig 2:**
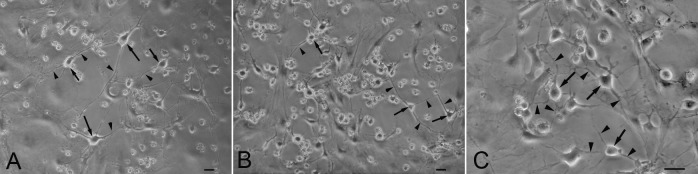
Isloated splenic TCs under phase-contrast microscope. **A.** Splenic TCs with typical morphology of piriform or triangular and small cell bodies and slender TPs; **B.** TCs forming a 3 D structure anchored by hetero- and homocellular junctions with their TPs; **C.** circle-like structure in TCs cultured in vitro, in which the long prolongations extended to support the wall of the circle-like structure. Scale bar = 20μm.

TCs were isolated and cultured *in vitro*. After plating the TCs adhere to the flasks in 60–90 min, attached TCs exhibited a small prominent body and their thin and long TPs after 44–48 hrs of incubation (Fig 2A–2C). TCs formed a three-dimensional network by their Tps connecting with other cells. The shape of telocytes changed at different time courses and telopodes changed from wide to thin. Telopodes extended and moved, leading to alternation of thin segments (podomers) and dilations (podoms). About 72 hrs later, some circle-like structures were detected, in which the long prolongations extended to support the wall of the circle-like structure (Fig 2D). TCs made up enough room for themselves and seemed like isolating themselves from other cells in the structure. Perhaps they could contribute to the formation of splenic hematopoietic niche and play important role in transmitting the signals.

### Live cell Imaging

**Fig 3:**
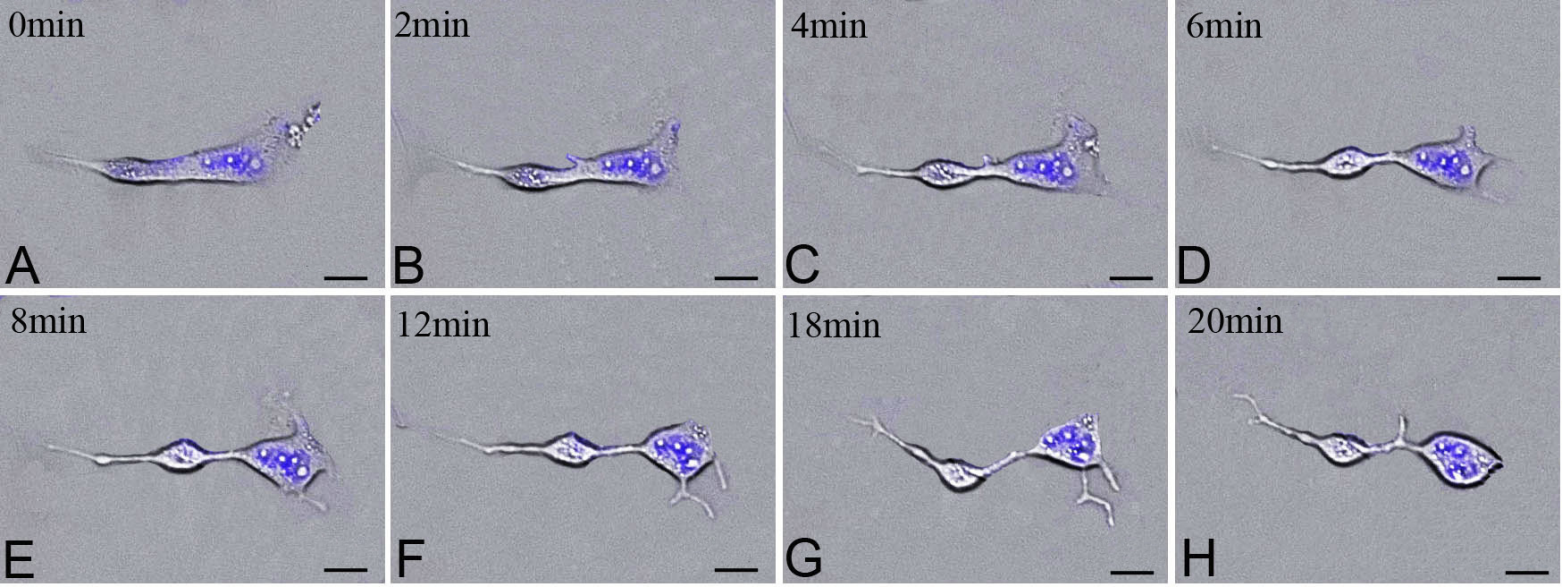
Dynamic morphology and movement of TCs captured by Live Cell Imaging System. Dynamic cytokinesis process in TCs under Live Cell Imaging System at 0 min in A, at 2 min in B, at 4 min in C, at 6 min in D, at 8 min in E, at 12 min in F, at 18 min in G, at 20 min in H, respectively; DAPI labeling nuclei of TCs excited with ultraviolet light(blue). Scale bar = 20μm.

Dynamic processes of proliferation and morphology in splenic TCs were observed under Live Cell Imaging System. Nuclei of TCs were labeled with DAPI, excited by ultraviolet light. So the dynamic processes were captured under white light and ultraviolet light. Merged images were showed in Fig 3. There were many mitosis phase in splenic TCs cultured in vitro. The intensity of nuclei increased during the process of DNA replication, then gradually reduced along with cytoplasmic separation. During cytokinesis, cytoplasm was pulled apart gradually and two new individual daughter cells came into being. One or more Tps were extended gradually and grown up in the form of two branches along with the cytoplasmic constriction. The duration of extending process was about 20 min and showed dynamic changes. Podoms were clearly observed and TCs could maintain their typical morphological features in different phases of mitosis.

### Scanning electron microscopy

**Fig 4:**
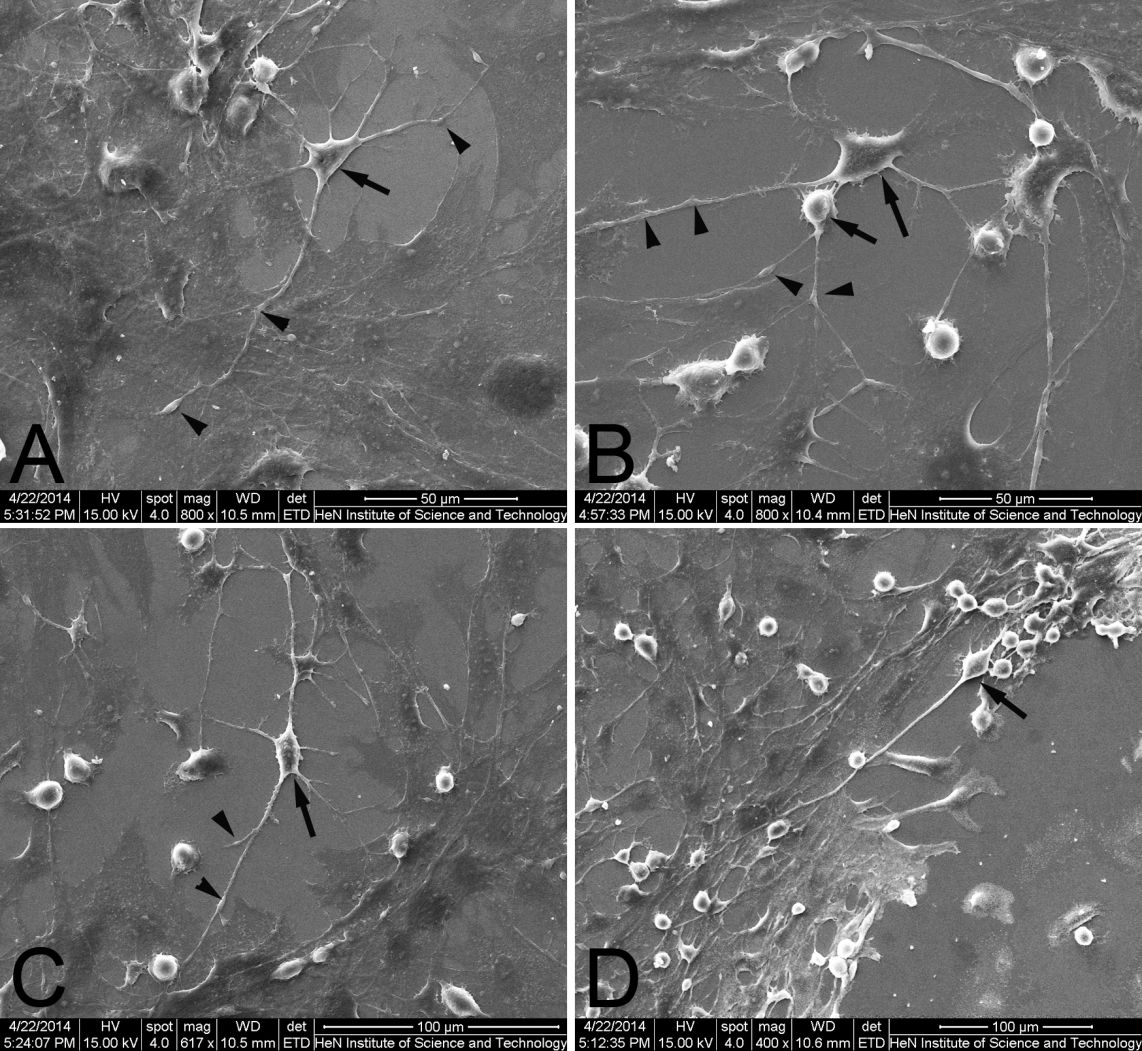
Isloated splenic TCs by scanning electron microscopy. **A.** An example of TC with 6 Tps. **B.** An example of two TCs keeping in touch with each other and growing their TPs with dichotomous branch out forming. **C.** An example of TC with TPs in different length (tens to hundreds of micrometres). **D.** An example of TC with two TPs and one of TPs reaching as 297.5μm. Black arrows indicate the piriform and small cell body and black arrowheads indicate the podoms arranged alternately on Tps.

**Fig 5:**
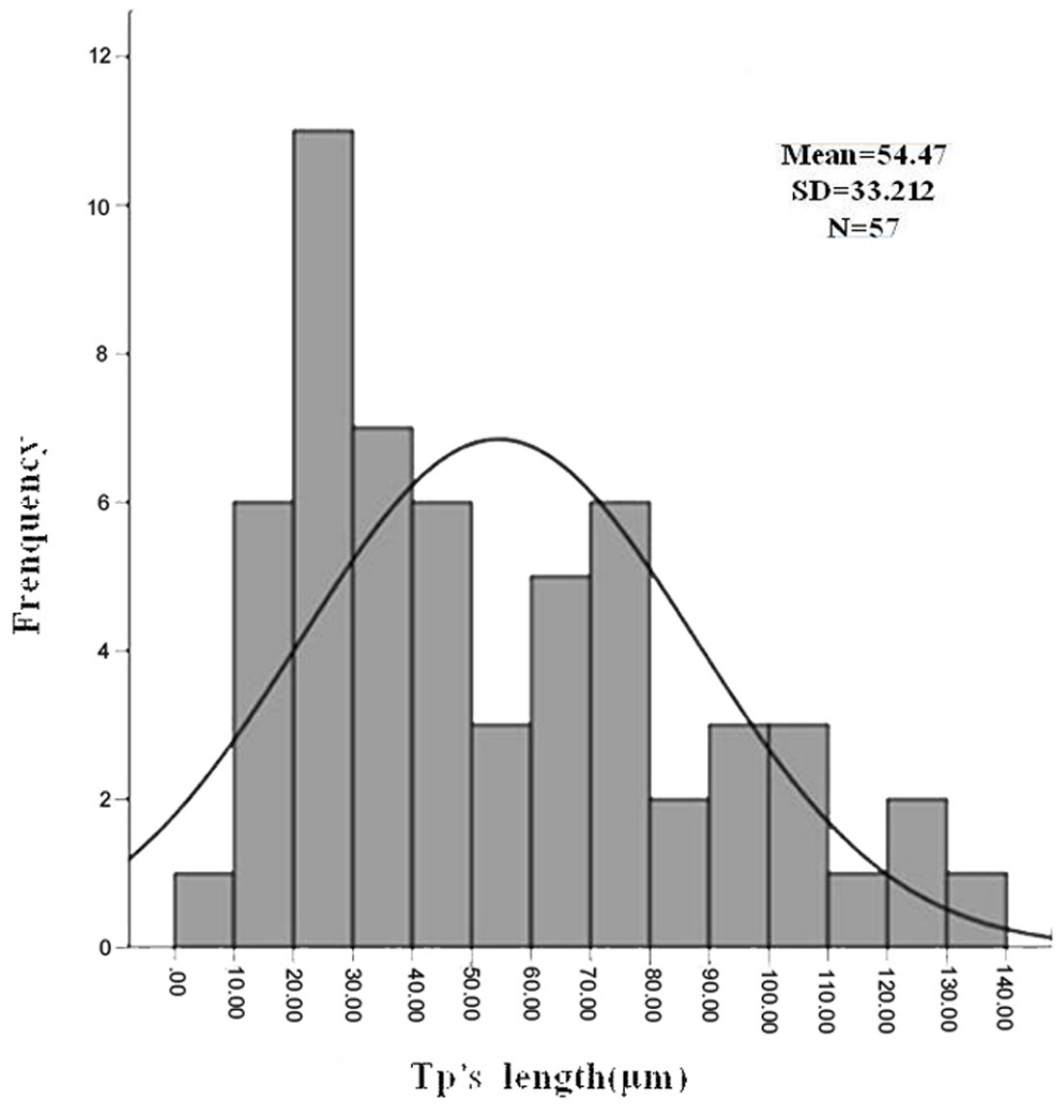
Positive skewed distribution of TP’s length.

The characterization of telocytes in the spleen was identified by SEM. Splenic TCs had a moniliform aspect with many dilations–a ‘bead on a string’ appearance (Fig 4). The thin, fibrillar segments are called podomers, and the dilated, cistern-like regions are called podoms (arrowheads indicate). TCs kept in touch with each other and grew their TPs with dichotomous branch out forming (Fig 4A and 4B). Splenic TCs displayed a small cell body (about 2–11 micrometers long, 0.1–4.6 micrometers wide and only 0.02–1.5 micrometers high) with slender shaped nucleus surrounded by a thin rim of cytoplasm and extended more than one TPs, from 9.93 μm to 133.67 μm (Fig 5). The length of their TPs was mostly distributing in 20–30 μm, in accordance with normal distribution (Fig 5). At the same time, total 310 telocytes were statistical analysed on number of TPs. The percentage of TCs was about 11.61% with only one TP, 22.58% with two TPs, 28.71% with three TPs, 18.06% with four TPs, 12.90% with five TPs, 3.23% with six TPs, 1.61% with seven TPs, 0.65% with eight TPs, 0.32% with ten TPs and 0.32% with eleven TPs respectively.

### Immunostaining of TCs

**Fig 6:**
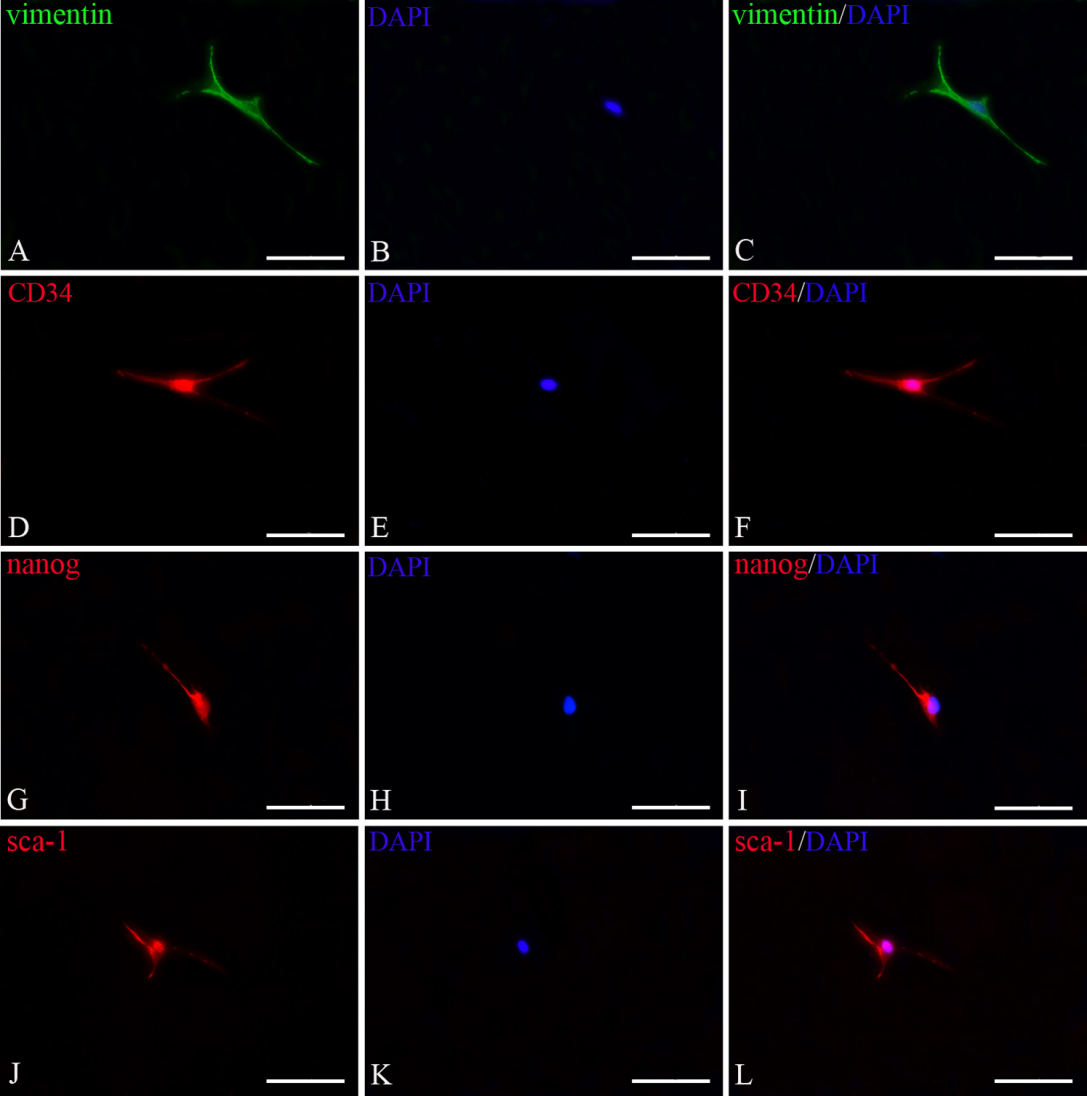
Detection of splenic TCs by immunofluorescence for vimentin, CD34, nanog and sca-1. **A.** Vimentin immunostaining (FITC fluorescence labeling, green); **D,G,J.** CD34, nanog and sca-1 immunostaining respectively (Cy3 fluorescence labeling, red); **B,E,H,K.** nuclei of TCs (DAPI staining, blue), **C.** merged image of A and B; **F.** merged image of D and E; **I.** merged image of G and H; **L.** merged image of J and K. Scale bar = 100μm.

**Fig 7:**
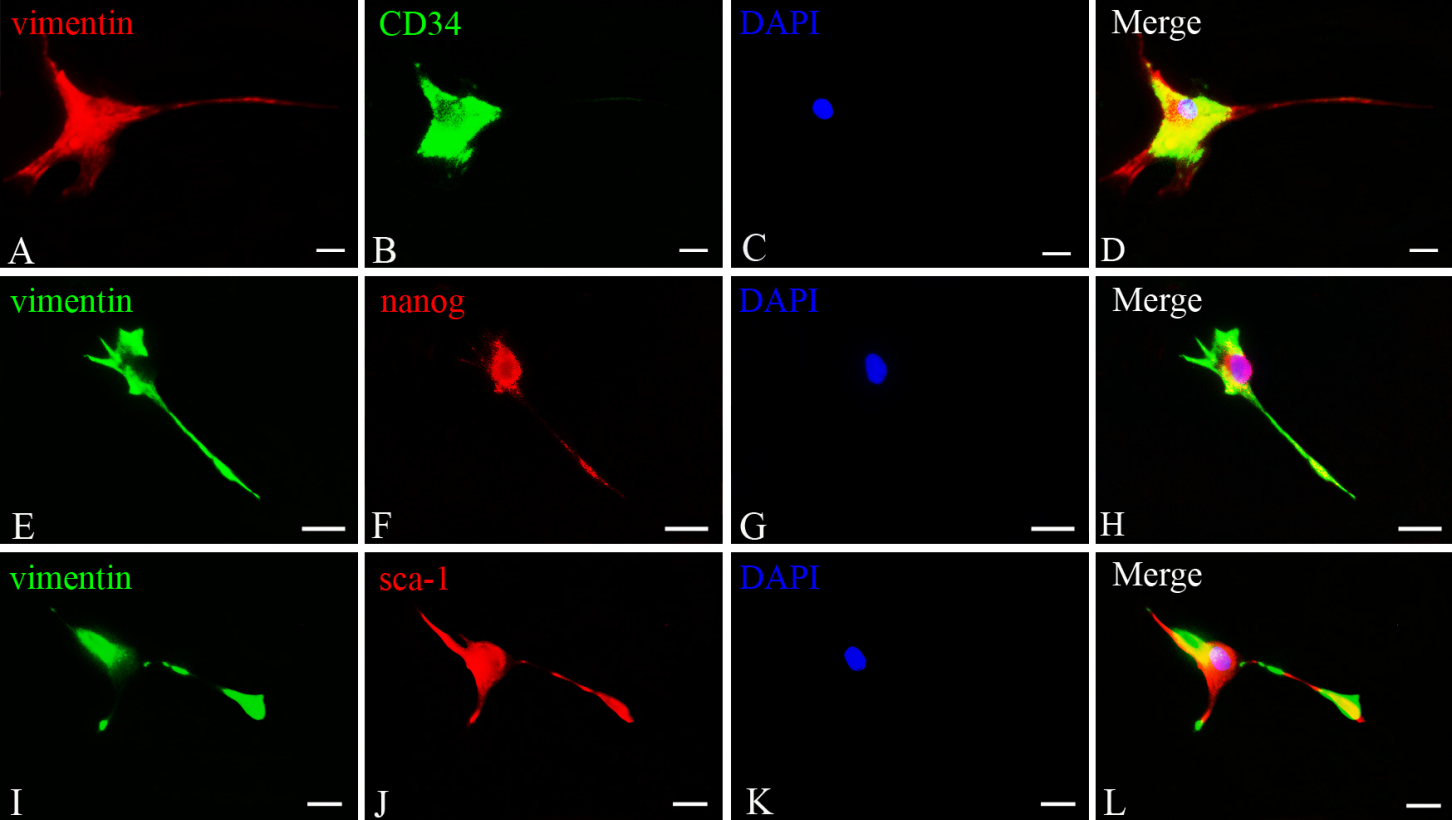
Double immunofluorescence staining for splenic TCs cultured *in vitro* A,E,I. Vimentin immunostaining (**A.** Cy3 fluorescence labeling, red; **E,I.** FITC fluorescence labeling, green); **B,F,J.** CD34, nanog and sca-1 immunostaining respectively (**B.** FITC fluorescence labeling, green; **F,J.** Cy3 fluorescence labeling, red); **C,G,K.** nuclei of TCs (DAPI staining, blue), **D.** merged image of A, B and C; **H.** merged image of E, F and G; **L.** merged image of I, J and K. Scale bar = 20μm.

TCs were positive for vimentin, the cytoskeletal component responsible for maintaining cell integritystabilizing cytoskeletal interactions, located in cell body and their prolongations (Fig 6). TCs also expressed CD34, nanog and sca-1. As shown in Fig 6, it appeared strongly positive for nanog and sca-1 in cell body and the initiation part of the prolongations. However, c-kit was negative in splentic TCs(Data not shown). Then double immunofluorescence staining to detect the TCs demonstrated there were co-expression of vimentin and CD34, vimentin and nanog, vimentin and sca-1 respectively (Fig 7). These phenotypes were stable following sub-culturing.

## Discussion

TCs are dispersed in the connective tissues, widely distributing in vertebrate organs [20]. With their long prolongations (TPs), TCs have homo- and heterocellular junctions and form three- dimensional networks. Abundant data show that TCs play important role in inter (trans)-cellular signaling [21, 22]. However, TCs and their roles in spleen remain unclear.

TCs are featured by their extremely long cellular prolongations termed telopodes (Tps). These Tps have an alternation of very thin segments named podomers and dilated portions named podoms [1,3,4]. Almost the features of TCs in various organs are consistent with small body and thin prolongations with a “beads on a string” appearance. In this study, splenic TCs and dynamic alterations of isolated and cultured TCs were detected. TEM images identified TCs and investigated their morphological and ultrastructural characteristics (Fig 1). Their contact with the surrounding cells in the spleen was also investigated. Consistent with the diagnostic criteria of TCs, splenic TCs were widely distributed in the interstitial spaces and were quite similar to those already reported in other organs [12]. They had one or more TPs in different length reaching tens to hundreds of micrometres and Tps mostly distributed in 20–30 micrometres (Fig 5). Statistical analysis on length of Tps indicated that the distribution of the frequency was positive skewed (Fig 5). Perhaps the samples were not large enough to be in accordance with normal distribution. Telocytes with three TPs and two TPs accounted for 28.71% and 22.58% respectively, which were the majority two types of telocytes in spleen. Tps had very thin, long and slender podomers interspersed with short podoms. Abundant mitochondria and endoplasmic reticulum were also detected in podoms (Fig 1). TCs kept in close touch with other TCs or leukomonocytes, including natural killer cells (NK), neutrophilicgranulocytes, macrophages, which were embraced with long prolongations of TCs.

Popescu *et al*. described these cells and had many comprehensive researches on TCs. They insisted on that no specific markers had been yet defined for TCs [3,4]. In the recent years, numerous reports have shown the existence of TCs in various mammalian cavitary and non-cavitary organs and have detected TCs in different tissues display different phenotypes [23, 24]. Cardiac TCs express vimentin, CD34, connectin 43 and PDGFR-β [25]. Cardiac TCs also express stem cell markers c-kit and sca-1 [26]. In skeletal muscle interstitium, TCs are found to express VEGF and PDGFR-β both *in situ* and *in vitro* [3, 27]. But it is usually accepted that CD34/PDGFRα and CD34/vimentin double-positive expression is appropriate to identify TCs [20]. However, immunostaining of TCs in spleen has not been understood. In our study, splenic TCs were found to express vimentin, CD34, nanog and sca-1, but they did not express c-kit. CD34 and Sca-1 are both used as phenotypic markers of relatively primitive hematopoietic progenitors [28, 29]. Nanog, one of the pluripotent stem cell markers, was firstly discovered in 2003 [30, 31]. Marker analysis of TCs in spleen has also led us to recognize their important roles. Our results demonstrate that splenic TCs have typical surface phenotypes, which suggesting some roles in the regenerative process. Further work is under way to obtain the full marker expression profile of splenic TCs, and to determine whether this cell type differs from that of other organs.

So far, the exact function of TCs has not been fully understood. Cardiac TCs were detected in stem cell niche in the vicinity of stem and progenitor cells [15, 17, 32]. TCs seem to provide support for cardiac stem cells (CSCs) in their niches. Our findings are consistent with these reports. We found some circle-like structure in TCs cultured in vitro, in which the long prolongations extended to support the wall of the circle-like structure. TCs usually leave enough room for themselves and seem like isolating themselves from other cells in the structure. Perhaps they could take part in formation of splenic hematopoietic niche and play important role in transmitting the signals. However, their exact role in the circle-like structure need to be further demonstrated.

Taken together, we have investigated the existence, characteristics, and distribution of TCs in the spleen and have detected the immunophenotypes of cultured TCs from the spleen, which could provide direct morphology evidence for exploring the exact role of TCs in spleen.
